# Swimming Training Induces Liver Mitochondrial Adaptations to Oxidative Stress in Rats Submitted to Repeated Exhaustive Swimming Bouts

**DOI:** 10.1371/journal.pone.0055668

**Published:** 2013-02-06

**Authors:** Frederico D. Lima, Daniel N. Stamm, Iuri D. Della-Pace, Fernando Dobrachinski, Nélson R. de Carvalho, Luiz Fernando F. Royes, Félix A. Soares, João B. Rocha, Javier González-Gallego, Guilherme Bresciani

**Affiliations:** 1 Laboratório de Bioquímica do Exercício, Centro de Educação Física e Desportos, Universidade Federal de Santa Maria (UFSM), Santa Maria, Rio Grande do Sul, Brazil; 2 Departamento de Química, Centro de Ciências Naturais e Exatas (CCNE), Universidade Federal de Santa Maria (UFSM), Santa Maria, Rio Grande do Sul, Brazil; 3 Institute of Biomedicine (IBIOMED) and Centro de Investigación Biomédica en Red de Enfermedades Hepáticas y Digestivas (CIBERehd), University of León, León, Spain; University of Valencia, Spain

## Abstract

**Background and Aims:**

Although acute exhaustive exercise is known to increase liver reactive oxygen species (ROS) production and aerobic training has shown to improve the antioxidant status in the liver, little is known about mitochondria adaptations to aerobic training. The main objective of this study was to investigate the effects of the aerobic training on oxidative stress markers and antioxidant defense in liver mitochondria both after training and in response to three repeated exhaustive swimming bouts.

**Methods:**

Wistar rats were divided into training (n = 14) and control (n = 14) groups. Training group performed a 6-week swimming training protocol. Subsets of training (n = 7) and control (n = 7) rats performed 3 repeated exhaustive swimming bouts with 72 h rest in between. Oxidative stress biomarkers, antioxidant activity, and mitochondria functionality were assessed.

**Results:**

Trained group showed increased reduced glutathione (GSH) content and reduced/oxidized (GSH/GSSG) ratio, higher superoxide dismutase (MnSOD) activity, and decreased lipid peroxidation in liver mitochondria. Aerobic training protected against exhaustive swimming ROS production herein characterized by decreased oxidative stress markers, higher antioxidant defenses, and increases in methyl-tetrazolium reduction and membrane potential. Trained group also presented higher time to exhaustion compared to control group.

**Conclusions:**

Swimming training induced positive adaptations in liver mitochondria of rats. Increased antioxidant defense after training coped well with exercise-produced ROS and liver mitochondria were less affected by exhaustive exercise. Therefore, liver mitochondria also adapt to exercise-induced ROS and may play an important role in exercise performance.

## Introduction

Exercise represents a physical stress that transiently disrupts homeostasis [1], and the working skeletal muscle is clearly the organ most directly affected during physical activity [2]. Studies indicate that exercise may induce structural damage to muscle cells [3], and the production of metabolic by-products, such as lactate [4], and reactive oxygen species (ROS) [5], [6]. There is consistent evidence that increased ROS production induced by acute intense exercise may cause an imbalance between oxidative intermediates and antioxidant systems, enhancing muscle lipid and protein oxidation, and the development of oxidative stress [7], [8], which was first defined by Helmut Sies in the 1980′s [9].

The metabolic adaptations to exercise are not restricted to the working muscle; exercise is also a major challenge to other organs such as cardiac muscle, stomach or brain [10], [11]. This is particularly relevant to the liver due to its central role in the maintenance of energy supply to the exercising muscle [12]. Studies aiming to evaluate the effects of the acute exercise on oxidative stress in the liver have shown increased lipid peroxidation [13]–[15], and protein carbonylation [16], [17], and decreased antioxidant defenses [18], [19]. Aerobic exercise performance demands energy supply which is mainly attended by increases in oxygen consumption. In the mitochondria, the oxygen consumed partially undergoes a one electron reduction, giving rise to the superoxide radical (O_2_^−^) [20], which is generated in different rates according to the assayed tissue [21]. Additionally, it is known that strenuous exercise causes a number of marked metabolic changes that may impair mitochondrial function in several ways [22], one major factor being mitochondrial ROS formation [23]. Interestingly, mitochondrial dysfunction appears to be a key issue during exhaustive exercise, and may cause oxidative damage and tissue injury to liver, among others organs [24].

ROS can also activate signal-transduction pathways to induce a stress-resistance response that protects against some of the toxic outcomes of ROS generation [25]. Indeed, exercise training has been reported to produce adaptive responses to oxidative stress, as studied primarily on skeletal muscles [26], but also in the liver. Thus, 8 weeks of aerobic training on treadmill increased the reduced/oxidized glutathione (GSH/GSSG) ratio [27], and 10 weeks of running upregulated superoxide dismutase (SOD) and catalase (CAT) liver enzyme activities in rats [28], [29]. Antioxidant capacity increases in SOD, CAT, glutathione reductase (GR), and GSH levels were also found after a 12-week exercise training in the liver of rats [30]. These experimental evidences point out to antioxidant regulation mechanisms in the liver driven by exercise training in rodents [12].

While different studies have investigated the response of the working skeletal muscle to acute exercise and training, considerably less is known about liver adaptations during and after increased physical activity [12]. Liver plays an important role during exercise through glucose release to the bloodstream and gluconeogenesis, and mitochondria are clearly important in exercise performance due to aerobic energy production. Of note, while exercise training seems to improve oxidative metabolism modulation, acute exercise bouts challenge the body’s antioxidant defenses with ROS production and exercise performance impairment. Competitive and tournament situations characterized by short recovery intervals between demanding exercise bouts may increase short-term ROS production confronting training adaptations [31]. In this sense, studies aiming to identify liver mitochondria adaptations to exercise-related oxidative stress in repeated stressful stimuli after training are still incipient. Therefore, the aim of this study was to evaluate the impact of swimming training on rat liver mitochondria oxidative stress modulation after training and repeated exhaustive swimming bouts.

## Materials and Methods

### Ethics Statement

The laboratory experiments were conducted in accordance with national and international legislations (Brazilian College of Animal Experimentation [COBEA] and the U.S. Public Health Service's Policy on Humane Care and Use of Laboratory Animals-PHS Policy) and approved by the Ethics Committee for Animal Research of the Universidade Federal de Santa Maria (UFSM; Permit number: 020848). Indeed, animal handling and laboratory assays were conducted in such a way that all efforts were made to minimize suffering.

### Animals and Reagents

Male Wistar rats (180–250 g) were kept in plastic boxes containing a maximum of five animals per cage, under controlled environment (12∶12 h light-dark cycle, with onset of light phase at 7∶00, 25±1°C, 55% relative humidity) with food (Guabi, Santa Maria, Brazil) and water *ad libitum*. Assay reagents were purchased from Sigma (St Louis, MO, USA).

### Study Design

**Figure 1:**
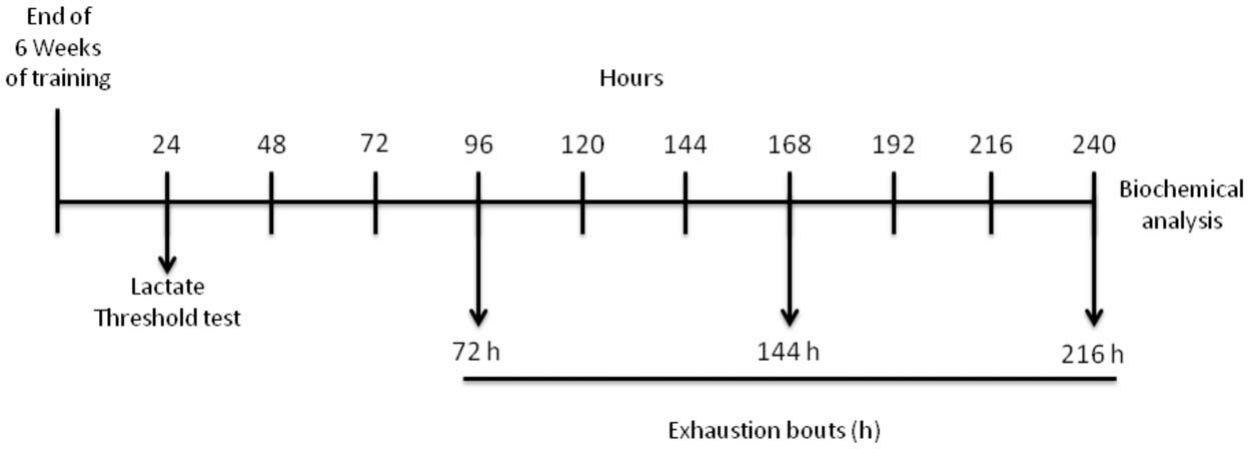
Timeline of the swimming training schedule and exhaustive protocol test data collection.

In this study animals were randomly divided into training and control groups. The training group performed a 6-week swimming training and 24 h after the last training session both groups performed a lactate threshold (LT) test. Subsets of control and training groups were sacrificed in order to assess training effects upon the biomarkers herein assayed. To study the effects of exhaustive exercise seventy two hours afterwards, rats from both groups performed 3 repeated exhaustive swimming bouts with each bout separated for a 72 h time period. Rats were sacrificed after the last bout and liver was immediately removed and prepared for mitochondria isolation. Antioxidant status, oxidative stress markers, and mitochondria potential viability were measured in liver mitochondria within different groups. Figure 1 depicts the study design.

### Water Adaptation

Rats were adapted to the water before the beginning of the experiment. The adaptation consisted on keeping the animals in shallow water at 31±1°C between 9∶00 to 11∶00 a.m. The adaptation period was carried out during the week before the swimming training onset. The purpose of the water adaption was to reduce stress without promoting physical training adaption.

### Training Protocol and Lactate Threshold Assay

The use of regular swimming exercise shows advantages over the treadmill protocol, because swimming is a natural ability of rats and is widely used [32]–[34]. For exercise training, animals were weighed and randomly assigned to the following groups: training and control. The training period consisted of 6 weeks, 60 min per day and 5 sessions per week. The tank used in this study was 80 cm in length, 50 cm in width and 90 cm in depth, and the swimming training was always performed in water temperature of 31±1°C (70 cm depth) between 10 to 12 h a.m. The training group begun the swimming training with a 5% body weight overload attached to the back to improve endurance [4]. The control group was placed in a separate but similar tank with shallow water (5 cm) at the same temperature for 30 min, 5 days a week without the back overload.

After 6 weeks of swimming training, a test protocol was used to determine the LT in control (n = 14) and training groups (n = 14). The LT test was carried out according to the protocol described by Marquezi et al. [35] with few modifications. The test consisted on 3 swimming bouts with progressive overload corresponding to 5%, 7% and 9% of each animal body weight for a period of 3 min for each load. A 1 min resting period was allowed between bouts. During the resting periods, 25 µl of blood were collected from the tail vein for lactate concentration assay, resulting in a total of 4 blood samples measured with a lactimeter (Accutrend® Plus, Roche Diagnostics GmbH, Germany). The LT for each animal was calculated based on the graphic inflection point when plotting lactate concentration against the corresponding exercise workload. Twenty four hours after the LT assay, control and trained animals subsets (n = 7) were killed by decapitation.

### Exhaustive Protocol Test

Three days after the LT test, an exhaustive protocol test was carried out according to de Araujo et al. [36] with few modifications. The protocol consisted in 3 repeated exhaustive swimming bouts: first bout took place 72 h after the LT test; second bout at 144 h after the LT test; and the third 216 h after the LT test (Figure 1). Animals swam individually in the tank with an overload of 13% of body weight until exhaustion in order to determine the time to exhaustion. Exhaustion was characterized by the moment at which animals were no longer able to maintain themselves in the water surface, reaching 10 s submerged [36]. When exhaustion was determined animals were taken out of the tank, dried and sacrificed.

### Mitochondrial Isolation

Liver mitochondria were isolated as previously described by Bhattacharya et al. [37], with few modifications. The liver was rapidly removed and immersed in ice-cold isolation buffer I (100 mM sucrose, 10 mM EDTA, 100 mM Tris–HCl, 46 mM KCl, at pH 7.4). The tissue was homogenized, and the resulting suspension was centrifuged for 3 min at 2000×g in a Hitachi CR21E centrifuge. After centrifugation, the supernatant was recentrifuged for 10 min at 12000×g. The pellet was resuspended in isolation buffer II (100 mM sucrose, 10 mM EDTA, 100 mM Tris–HCl, 46 mM KCl, and 0.5% bovine serum albumin (BSA) free of fatty acids, at pH 7.4) and recentrifuged at 12000×g for 10 min. The supernatant was decanted, and the final pellet was gently washed and resuspended in 125 µl of isolation buffer III (270 mM mannitol, 70 mM sucrose, 0.02 mM EDTA, 20 mM Tris–HCl, 1 mM K_2_HPO_4_, at pH 7.4).

### Reduced (GSH) and Oxidized Glutathione (GSSG) Content

GSH and GSSG levels were determined with fluorescence detection after reaction of the supernatants from deproteinized mitochondria containing H_3_PO_4_/NaH_2_PO_4_–EDTA or H_3_PO_4_/NaOH, respectively, with *O*-phthalaldehyde (OPT) [38]. In brief, freshly isolated liver mitochondria (0.5 mg prot/ml) resuspended in 1.5 ml phosphate buffer (100 mM NaH_2_PO_4_, 5 mM EDTA, pH 8.0) and 500 µl H_3_PO_4_ 4.5% were rapidly centrifuged at 100000×g (Hitachi, TL-100 ultracentrifuge) for 30 min. For GSH determination, 100 µl of supernatant was added to 1.8 ml phosphate buffer and 100 µl OPT. After thorough mixing and incubation at room temperature for 15 min, the solution was transferred to a quartz cuvette and the fluorescence was measured at 420 and 350 nm emission and excitation wavelengths, respectively. For GSSG determination, 250 µl of the supernatant was added to 100 µl of *N*-ethylmaleimide and incubated at room temperature for 30 min. After the incubation, 140 µl of the mixture was added to 1.76 ml NaOH (100 mM) buffer and 100 µl OPT. After mixing and incubation at room temperature for 15 min, the solution was transferred to a quartz cuvette and the fluorescence was measured at 420 and 350 nm emission and excitation wavelengths, respectively. GSH and GSSG contents were determined from comparisons with a linear GSH or GSSG standard curve, respectively.

### Manganese Superoxide Dismutase (MnSOD) Activity

The MnSOD enzyme activity was determined in liver mitochondria according to the method proposed by Misra and Fridovich [39]. This method is based on the capacity of MnSOD in inhibiting autoxidation of adrenaline to adrenochrome. In brief, the supernatant fraction (100 µl) was added to a medium containing sodium bicarbonate–carbonate buffer (50 mM; pH 10.2) and adrenaline (0.4 mM). The kinetic analysis of MnSOD was started after adrenaline addition, and the color reaction was measured at 480 nm.

### Thiobarbituric Acid Reactive Substances (TBARS) Levels

Lipid peroxidation was estimated by measuring TBARS according to the method of Ohkawa et al. [40], [41]. In this method, malondialdehyde (MDA), an end product of fatty acid peroxidation, reacts with thiobarbituric acid (TBA) to form a colored complex. In brief, the supernatant fraction of liver mitochondria was incubated at 100°C for 60 min in acid medium containing 8.1% sodium dodecyl sulfate, 0.5 ml of acetic acid buffer (500 mM, pH 3.4) and 0.6% TBA. TBARS levels were measured at 532 nm, and expressed as nmol TBARS/mg mitochondrial protein.

### Protein Carbonyl Levels

Protein oxidation in liver mitochondria was measured as concentration of protein carbonyls formed, and the levels were determined using 2,4 dinitrophenylhydrazine (DNPH) assay [42]. The mitochondria were divided into two portions containing 1 mg protein/ml each. To one portion, 1 ml of 2 N HCl was added and incubated at room temperature shaking intermittently for 1 h. The other portion was treated with 1 ml of 10 mM DNPH in 2 N HCl and incubated by shaking intermittently for 1 h at room temperature. After incubation the mixture was precipitated with 10% TCA and centrifuged. The precipitate was washed three times with 1 ml of ethanol:ethyl acetate (1∶1). The final protein precipitate was dissolved in denaturation buffer (3% SDS and 150 mM NaH_2_PO_4_; pH 6.8) and the absorption at 370 nm (DNPH-treated sample minus sample blank) was determined. Carbonyl content was calculated using the molar extinction coefficient of 22,000 and expressed as nmol DNPH/mg mitochondrial protein.

### Methyl-Tetrazolium (MTT) Reduction Levels

MTT assays were carried out with a modification [43] of the method described by Berridge and Tan [44], except that the respiration buffer was used as the medium. MTT reduction levels were determined as an index of the dehydrogenase enzymes functions, which are involved in the cellular viability. Samples were incubated in buffer containing glutamate/succinate (5 mM each) and MTT (0.5 mg/ml) for 30 min at 37°C, and MTT reduction reaction was stopped by the addition of 1 ml of dimethylsulphoxide (DMSO). The formed formazan levels were determined spectrophotometrically, reported as the difference in absorbance between 570 and 630 nm and the results were corrected by the protein content. Individual samples were expressed as a percent of the mean control value in the experiment.

### Mitochondrial Membrane Potential (*Δψ*) Determination

The mitochondrial *Δψ* determination was assayed according to Akerman and Wikstron [45]. Brieﬂy, the mitochondria samples (150 µg protein/ml) were incubated in a medium containing KCl (65 mM), sucrose (100 mM), EGTA (0.05 mM), BSA (0.2%), HEPES (10 mM, pH 7.2), safranine O (10 µM) and the respiratory substrates glutamate (5 mM) and succinate (5 mM). The reaction was started with the mitochondria addition and the medium was kept at constant stirring during the assay period. The ﬂuorescence analysis was performed at 495 nm for excitation and 586 nm for emission, with slit widths of 5 nm. Results are presented as arbitrary units of fluorescense units per second relative to % control.

### Estimation of ROS Production

Production of ROS was estimated in liver mitochondria with the fluorescent probe, 2′,7′-dichlorofluorescein diacetate (DCFH-DA), as described by [46], [47]. Briefly, tissues were homogenized in 2.5 ml of saline solution (0.9% NaCl). Aliquots of 2.5 ml were incubated in the presence of DCFH-DA (5 µm) at 37°C for 60 min. The DCFH-DA is enzymatically hydrolyzed by intracellular esterases to form nonfluorescent DCFH, which is then rapidly oxidized to form highly fluorescent 2′,7′-dichlorofluorescein (DCF) in the presence of ROS. DCF fluorescence intensity is proportional to the amount of ROS that is formed. Fluorescence was measured using excitation and emission wavelengths of 480 and 535 nm, respectively. A calibration curve was established with standard DCF (0.1 nm to 1 µm), and ROS levels were expressed as percentages of control.

### Protein Determination

The protein content was measured colorimetrically by the method of Bradford [48] using bovine serum albumin (1 mg/ml) as standard.

### Statistical Analysis

The Statistical Package for Social Sciences (SPSS, Ins, Chigaco, IL) version 17 was used for all analyses. Data were expressed as mean ± standard error of means (SEM). Significance was assessed by one- or two-way analysis of variance (ANOVA), followed by Newman–Keuls’s Test for post-hoc comparison when appropriate. Statistical significance was set at p<0.05.

## Results

### Swimming Training Effects on Lactate Threshold, Exhaustion time, and Body Weight

**Figure 2:**
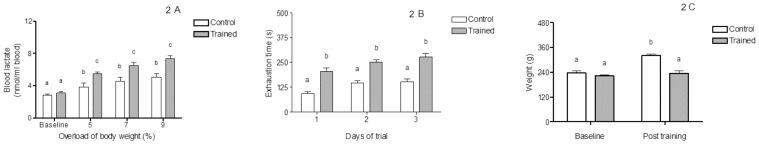
Effect of 6 weeks of swimming training on lactate threshold, time to exhaustion, and body weight. (A–C) Values are mean ±SEM (n = 14). Means without a common letter differ, p<0.05.

Lactate threshold, exhaustion time, and body weight were previously demonstrated to be altered by swimming training [49]–[51]. Statistical analysis revealed that blood lactate concentration increased progressively for both trained and control rats, though the trained group presented lower lactate concentrations comparing to the control group [F(1,12) = 23.41; p<0.05; Fig. 2A]. The trained group showed a significantly higher time to exhaustion in comparison to the control group in day 1 [F(1,14) = 33.68; p<0.05; Figure 2B], day 2 [F(1,14) = 42.57; p<0.05; Figure 2B] and day 3 [F(1,14) = 27.71; p<0.05; Figure 2B] of the trial. A significant increase in total body weight of control rats comparing to trained rats was observed after the 6 weeks of swimming training [F(1,12) = 34.58; p<0.05; Figure 2C].

### GSH and GSSG Content, and GSH/GSSG Ratio

**Figure 3:**
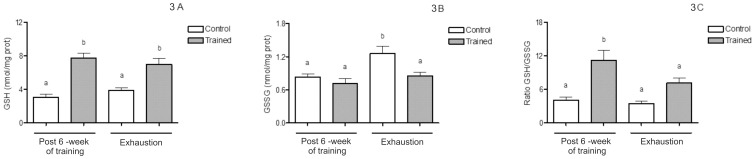
GSH, GSSG, and GSH/GSSG ratio levels both after 6 weeks of swimming training and an exhaustive protocol test. (A–C) Values are mean ± SEM (n = 7). Means without a common letter differ, p<0.05. GSH: reduced glutathione; GSSG: oxidized glutathione.

The trained group showed significantly higher liver mitochondria GSH levels [F(1,20) = 52.56; p<0.05; Figure 3A] and GSH/GSSG ratio [F(1,20) = 32.01; p<0.05; Figure 3C] comparing to the control group after 6 weeks of swimming training. The exhaustive exercise protocol test also resulted in increased GSH levels and GSH/GSSG ratio in the trained group, while GSSG levels were significantly higher in the control group [F(1,20) = 9.39; p<0.05; Figure 3B].

### MnSOD Activity, and TBARS and Protein Carbonyl Levels

**Figure 4:**
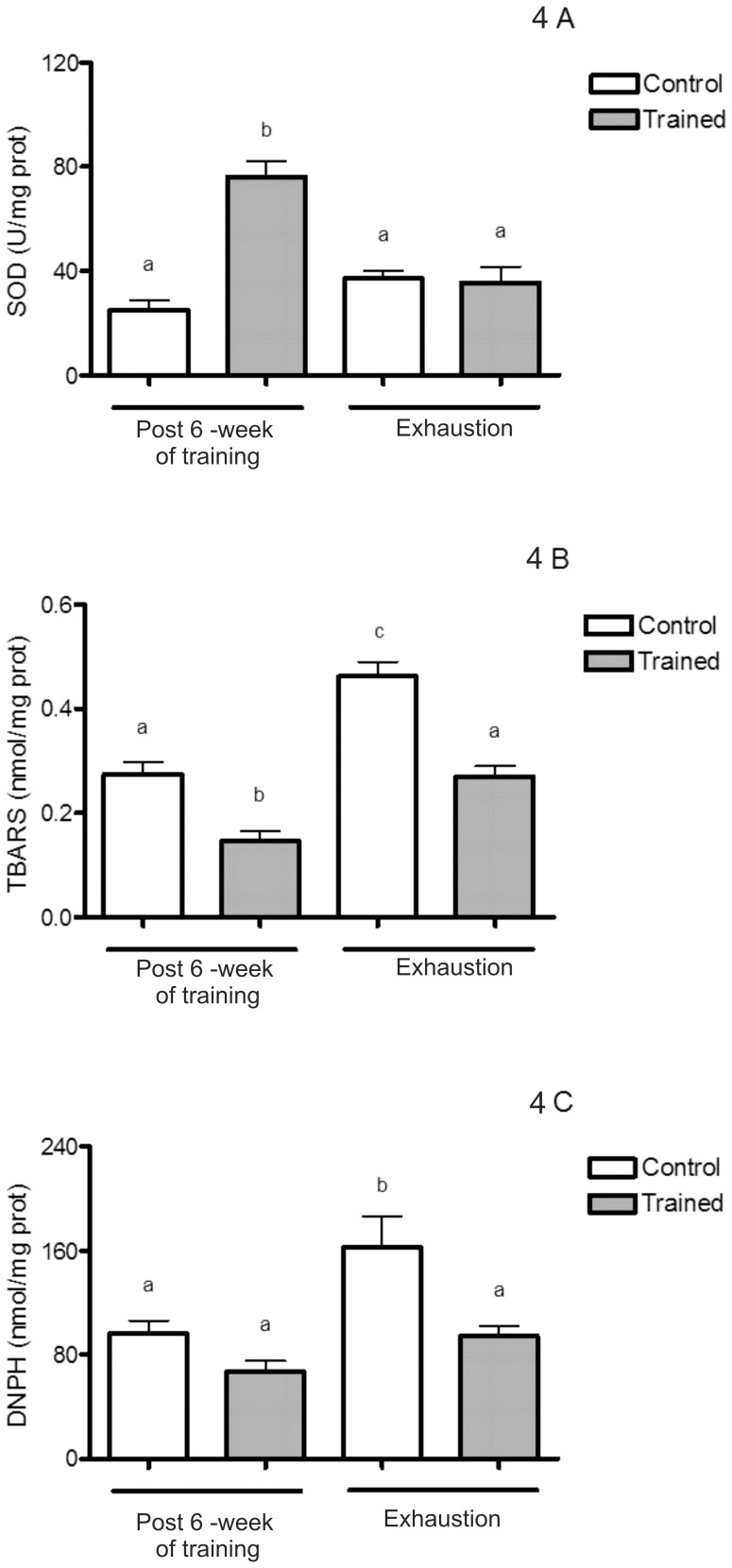
MnSOD activity, TBARS and protein carbonyls levels both after 6 weeks of swimming training and an exhaustive protocol test. (A–C) Values are mean ±SEM (n = 7). Means without a common letter differ, p<0.05. MnSOD: manganese superoxide dismutase; TBARS: thiobarbituric acid reactive substances; DNPH: dinitrophenylhydrazine.

The statistical analysis showed that swimming training increased MnSOD activity [F(1,29) = 22.97; p<0.05; Figure 4A] and decreased TBARS level [F(1,30) = 45.70; p<0.05; Figure 4B] in comparison to control rats. Training prevented TBARS [F(1,30) = 42.52; p<0.05, Figure 4B] and protein carbonyl increases [F(1,22) = 14.66; p<0.05 Figure 4C] induced by the exhaustive exercise protocol in the control group, suggesting the aforementioned GSH upregulation and MnSOD increase with training may protect against TBARS and protein carbonyl increase after the exhaustive protocol test.

### Mitochondria MTT Reduction and Membrane Potential, and ROS Production

**Figure 5:**
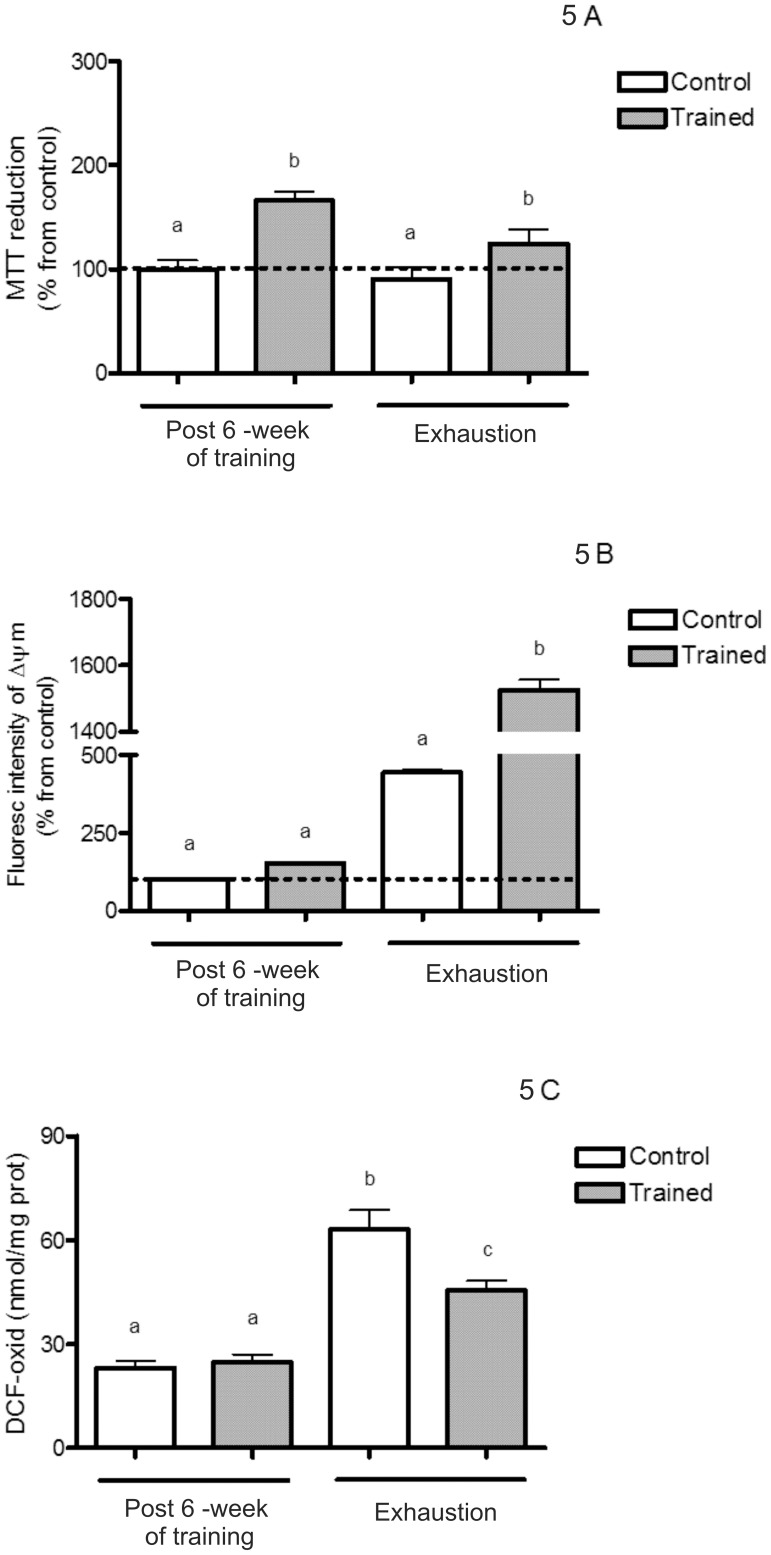
MTT reduction and membrane potential, and ROS production both after 6 weeks of swimming training and an exhaustive protocol test. (A–C) Values are mean ±SEM (n = 7). Means without a common letter differ, p<0.05. ROS: reactive oxygen species; MTT: Methyl-tetrazolium; DCF-oxid: oxidized dichlorofluorescein diacetate; *Δψ*: membrane potential.

Statistical analysis showed that training resulted in a higher liver mitochondria MTT reduction both after training and to the exhaustive protocol test in comparison to the control group [F(1,26) = 25.90; p<0.05; Figure 5A]. On the same line, the *Δψ* also increased in trained group after the exhaustive protocol test [F(1,27) = 16.94; p<0.05; Figure 5B]. The exhaustive protocol test induced increases in DCFH oxidation both in trained and control rats, with values significantly lower for trained animals [F(1,26) = 83.95; p<0.05; Figure 5C].

## Discussion

In the current study we have shown a liver mitochondria adaptive response to exercise training characterized by increases in GSH/GSSG ratio, MnSOD activity, mitochondrial viability and *Δψ*, and decreases in TBARS levels in trained animals. To the best of our knowledge this is the first study to point out that swimming training protects against liver mitochondria oxidative damage after repeated bouts of forced swimming. The results presented in this report also support the hypothesis that acute exercise causes oxidative and mitochondrial stress [17], with increased TBARS and protein carbonyl levels and higher ROS production after an exhaustive exercise bout. These experimental findings suggest that acute exercise causes mitochondrial ROS generation and that this oxidative disruption may benefit by activating antioxidant defense systems during exercise training programs.

We found a body weight plateau in trained rats during the 6 week training period when compared to the control group. Other authors have also reported that swimming training stabilizes body weight in rats [52], [53]. According to Ravi Kiran et al [52], who investigated different training intensities and durations in 4 and 12 months-old rats, the training protocol intensity applied in our study can be considered as high intensity (5% overload, 60 min/day –5 days/week). A high intensity swimming protocol demands high energy matching capacities so as to overcome the task. Body weight reduction in humans promoted by high-intensity exercise training originates from intense lipid usage during recovery periods following high-intensity glycogen-depleting exercise [54]. When exercise results in glycogen depletion, muscle glycogen resynthesis is of high metabolic priority, resulting in the preferential use of intramuscular triacylglycerol and circulating lipids by the recovering skeletal muscle [55]. These same mechanisms also seem to take place in rats [28]. In accordance, in our study lactate level was lower and time to exhaustion was higher in the trained group, corroborating previous findings [36], [49]–[51].

Concerning antioxidant effects of exercise, a substantial body of evidence suggests that regular exercise plays an important preventive and therapeutic role in oxidative stress-associated diseases, including ischemic heart disease, type II diabetes, and Alzheimer’s disease [56]–[58]. Accordingly, studies have shown that animals and humans clearly undergo significant adaptive responses to regular endurance exercise that involve greatly increased endurance capacity, which is permitted by dramatic mitochondrial biogenesis, reduction in oxidant production and increased antioxidant defenses [59], [60]. In this context, the liver plays a key role in exercise-induced oxidative stress. For instance, liver is the major organ for *de novo* GSH synthesis, supplying 90% of the circulating GSH, which is one of the most important endogenous antioxidants [61] and plays an important role as a reducing agent [62], protecting the organism against hydrogen peroxide (H_2_O_2_) and lipid peroxides [63]. Sun et al. [61] found increased liver mitochondria GSH after 4 weeks of endurance training in rats, which was attributed to an increased antioxidant activity. Navarro et al. [64] also reported that chronic moderate exercise increases MnSOD activity and decreases mitochondrial oxidation products (TBARS and protein carbonyls) in trained rat liver, suggesting that changes were consistent with a faster mitochondria turnover and biosynthesis.

In the present study we found an increase in MnSOD activity and GSH/GSSG ratio, concomitant with decreased TBARS and protein carbonyl levels in trained rats. On the same line, GSH level presented a 2-fold increase after training and levels were maintained after the exhaustive protocol test. This is a remarkable finding since GSH depletion in cells is involved in metabolic limitations such as lower exercise capacity and cell membrane disruption/apoptosis that may lead to oxidative stress [18]. In agreement to this view, Botezelli et al. [28] have recently demonstrated that 8 weeks of swimming training decreased lipid peroxidation, a fact partially attributed to an improved antioxidant system with greater SOD enzyme activity. Our data show the same tendency, with decreased TBARS levels and higher MnSOD activity in trained rats comparing to the control group, suggesting that the MnSOD activity increase in liver mitochondria may be an antioxidant response to the oxidative injury caused by exercise. Together with higher GSH levels, augmented MnSOD activity after training may cope with ROS production and thus prevent from lipid peroxidation and further oxidative stress. These experimental findings indicate a clear adaptation of liver mitochondria towards an enhanced antioxidant system after swimming training.

Considering that several expressed enzyme systems contribute to ROS formation in the liver and that SOD efficiently removes excessive ROS to maintain the normal cell homeostasis [65], it is plausible to propose that biochemical training adaptation as upregulation of antioxidant enzymes (MnSOD) reflects on decreased markers of lipid and protein peroxidation. Therefore, the increased mitochondrial chain respiratory function (characterized here by mitochondrial electron flow and *Δψ*) in trained rats suggests that mitochondrial redox status elicited by exercise-related oxidative stress may influence on a long lasting exercise performance. Our data showed that the trained group presented diminished TBARS content and higher MnSOD activity comparing to the control group. Swimming training enhanced antioxidant defenses to repeated exhaustive swimming bouts and enforced antioxidant defenses to adapt to these repeated stimuli.

Elevated TBARS levels after the exhaustive protocol test indicate increased membrane lipid peroxidation in the liver [16]. On the same line, ROS interaction with enzymes and structural proteins may cause thiol oxidation and protein carbonyls introduction, affecting activity and function of such molecules [66], [67]. In the present study we found a significant increase in TBARS and protein carbonylation in the control group of animals after 3 exhaustive swimming bouts. Liu et al. [17] also reported an increase in liver TBARS levels of rats submitted to an acute exercise bout. An interesting finding in our research is that swimming training prevented TBARS and protein carbonyls increases after the exhaustive protocol test in comparison to the control group. It has been shown that increased TBARS and protein carbonyls in the mitochondria membranes impair membrane-bound enzyme activities leading to mitochondrial dysfunction [64]. These data suggest that swimming training may provide protection to acute insult in the liver mitochondria herein measured by oxidative stress markers. In this context, our data show an adaptation of liver mitochondria towards an enhanced antioxidant system after swimming training.

Considering the energy supply by mitochondria, its dysfunction appears to play a key role in exercise performance [68]. We isolated liver mitochondria to demonstrate MTT reduction, *Δψ*, and ROS production across DCFH oxidation. The MTT reduction depends on the functionality of the oxidoreductase enzyme pool, such as the dehydrogenases [44]. Considering most of these are mitochondrial enzymes [44], [69], functional impairment may be related to mitochondrial functional impairment. In this sense, experimental findings revealed that swimming training increased MTT reduction and this step up was associated with higher *Δψ* and lower increases on ROS production after the exhaustive protocol test comparing to the control group. On the same line, the control group registered higher ROS production after the exhaustive exercise protocol. Of note, the trained group presented higher time to exhaustion during the exhaustive protocol test. Taken together these findings suggest that liver mitochondria dysfunction may be related to exercise-induced fatigue in the control group. It is plausible to suggest a mitochondrial role on exercise-induced fatigue considering that muscle mitochondria are responsible for metabolic stability improvement following endurance training [70].

Noteworthy a major mitochondrial enzyme here analyzed was dampened by the exhaustive protocol test. Our data suggest that MnSOD inhibition combined with higher DCFH oxidation after the exhaustive protocol test may be related to accumulative stress produced across three exhaustive swimming bouts, as indicated by high TBARS and protein carbonyls levels in the control group. In line with this view, we found that swimming training induced a significant increase in liver mitochondrial GSH content, MnSOD activity, and MTT reduction in the trained group, suggesting that ROS production is of key importance in the modulation of signaling pathways involved in the liver adaptation to exercise training. In fact, H_2_O_2_, the byproduct of O_2_^−^ dismutation, has been reported to activate several signaling pathways across interactions with different molecules [25]. Data herein presented confirm this hypothesis, with trained rats showing higher antioxidant status, enhanced antioxidant enzyme activity, lesser mitochondrial oxidation, and increased mitochondrial viability.

It has been described that exercise training produces remarkable changes in liver metabolism [13], [17], [19]. Even though we did not characterized the hepatocytes prior to mitochondria isolation, a recent study using a similar swimming protocol (8 weeks, 1 h/day –5.2% body weight overload) showed no differences in shape and size of hepatocytes and nuclei, and no changes in hepatic protein/DNA ratio (a marker of hyperplasia in the liver) after swimming training both in diabetic and normal rats [71]. Moreover, the swimming training applied did not induce changes in the amount of mitochondria present in liver cells or the mitochondria matrix [71]. Another issue that may be addressed is the possible difference in total liver parameters compared to isolated mitochondria measurements. Although we did not perform total homogenate measurements, previous reports have failed to find significant differences among tissue and isolated mitochondria. Thus, no differences between GSH and TBARS in tissue homogenate and isolated mitochondria have been observed following endurance exercise in rat [61].

In summary, the present study reports that swimming training induces positive adaptations in liver mitochondria of rats, characterized by increases on GSH/GSSG ratio, MnSOD activity, MTT reduction, *Δψ*, and decreases on TBARS and protein carbonyl levels. For the first time a response to accumulative exercise-induced stress was reported in liver mitochondria, with control rats presenting higher ROS production associated with an increased TBARS and protein carbonyl levels after repeated exhaustive swimming bouts. On the other hand, increased antioxidant defense induced by swimming training coped well with exercise-produced ROS and thus preserved liver mitochondria redox status after the exhaustive protocol test. Data showing specific molecular systems modulation by physical exercise also provide a framework to guide further studies aimed to examine mechanisms by which regular exercise may alter hepatic mitochondrial metabolism and protect against exercise-induced stress. Considering the importance of liver mitochondria in energy supply and antioxidant defenses, it becomes clear that this organelle may play a role in exercise performance and further studies to determine the participating signaling pathways are of interest.
